# Maximizing energy coupling to complex plasmonic devices by injecting light into eigenchannels

**DOI:** 10.1038/s41598-017-10148-w

**Published:** 2017-08-29

**Authors:** Yonghyeon Jo, Wonjun Choi, Eunsung Seo, Junmo Ahn, Q-Han Park, Young Min Jhon, Wonshik Choi

**Affiliations:** 1 Center for Molecular Spectroscopy and Dynamics, Institute for Basic Science, Seoul, 02841 Korea; 2 Department of Physics, Korea University, Seoul, 02841 Korea; 3 Sensor System Research Center, Korea Institute of Science and Technology, Seoul, 02792 Korea

## Abstract

Surface plasmon polaritons have attracted broad attention in the optoelectronics field due to their ability to merge nanoscale electronics with high-speed optical communication. As the complexity of optoelectronic devices increases to meet various needs, this integration has been hampered by the low coupling efficiency of light to plasmonic modes. Here we present a method to maximize the coupling of far-field optical waves to plasmonic waves for arbitrarily complex devices. The method consists of experimentally identifying the eigenchannels of a given nanostructure and shaping the wavefront of incident light to a particular eigenchannel that maximizes the generation of plasmonic waves. Our proposed approach increases the coupling efficiency almost four-fold with respect to the uncontrolled input. Our study will help to facilitate the integration of electronics and photonics.

## Introduction

Surface plasmon polaritons (SPPs), collective charge oscillations on conducting material induced by electromagnetic radiation, have attracted broad research interest over the past decades due to interesting properties such as a lateral field confinement that is well below the size of their wavelength and their propagation as surface waves. These features are responsible for counter-intuitive phenomena, such as the extraordinary transmission of far-field waves through tiny nanoholes^1^, and enable SPPs to be used in highly sensitive bio-sensing^2–4^. In recent years, a large volume of research has been conducted with the goal of producing plasmonic integrated circuits for high-speed optical communication networks at the subwavelength scale of electronics platforms^5^. Many of these studies have looked to fabricate the building blocks of integrated circuits, such as nanolight source^6,7^, waveguides^8,9^, switches^10,11^, couplers^12,13^, modulators^14,15^, and logic gates^16,17^.

In many cases, plasmonic devices use band-gap structures to guarantee the optimal coupling of far-field waves to SPPs^12,18^. Otherwise the coupling efficiency is so low that only a very small fraction of the excitation energy can be used. However, the working conditions for these band-gap structures are so strict that the wavelength, polarization, and angle of illumination need to remain fixed to ensure the best performance. These stringent conditions limit the design freedom of integrated circuits that host multiple functionalities because they can cause a conflict between the implementation of the function and the excitation of the SPPs. To solve this problem, devices with two or more band-gap structures have been proposed, meaning they can couple light to multiple wavelengths^19,20^ or polarizations^18^. While this approach holds great promise for promoting an increase in the complexity of devices, the coupling efficiency drops as the number of band-gap structures increases.

In this letter, we present a systematic method to maximize the coupling of far-field waves to SPPs for complex metallic nanostructures that do not have band-gap structures. In an experimental demonstration, a disordered arrangement of multiple slits was prepared on thin Au film. We exploit the approach used in complex nanophotonics in which the knowledge of the transmission matrix of a highly disordered medium allows wave propagation through the medium to be controlled^21–25^. In our study, we experimentally constructed a transmission matrix for the disordered nanoslits by measuring complex field maps of the SPPs for the excitation of each far-field mode. By performing the singular value decomposition of this unique transmission matrix, we identified the eigenchannels with large singular values. The incident far-field wave then had its wavefront shaped to couple to these eigenchannels. By doing so, the coupling efficiency was observed to increase by almost four times when compared to uncontrolled inputs. Since the injection of light waves to input channels other than eigenchannels leads to the generation of weaker SPPs than the eigenchannel coupling case, our method is a systematic procedure to maximize the coupling of far-field waves to the SPPs for any given nanostructures.

We also conducted a theoretical analysis to confirm the validity of the proposed method. The coupled mode method (CMM) based on modal expansion formalism^26^ was extended to incorporate the control of the wavefront of an incident wave. For a double-slit structure, which is simple enough for the extended CMM to handle, we verified that the eigenchannels identified from the recorded transmission matrix were in excellent agreement with the theoretical prediction.

### Experimental setup for the construction of the transmission matrix

**Figure 1:**
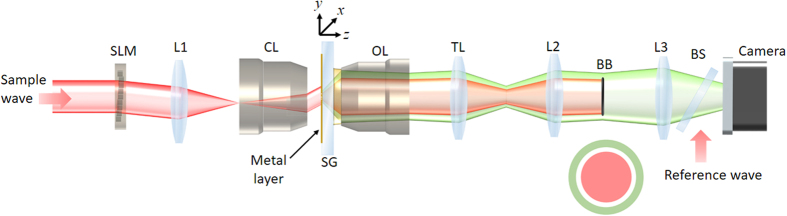
Schematic experimental setup. SLM: spatial light modulator (Hamamatsu Photonics, X10468–06). CL: condenser lens (Nikon, numerical aperture: 1.4). OL: objective lens (Nikon, numerical aperture: 1.4). SG: slide glass. BB: beam block. BS: beam splitter. Camera (RedLake M3). L1, L2, L3, and TL: lenses. A metal layer with nanostructures was coated on the slide glass. The output beam from a He-Ne laser (not shown) was split into sample and reference waves and these were later combined at the BS to form an interference pattern in the camera. The far-field waves are indicated in red and the SPPs generated at the metal layer are indicated in green.

We constructed a leakage radiation microscope^27,28^ for the wide-field detection of SPPs and added a reference arm to measure the phase and amplitude maps of the detected SPPs (Fig. 1). An output beam from a He-Ne laser (wavelength λ = 633 nm) was split into sample and reference waves, and a spatial light modulator (SLM: Hamamatsu Photonics, × 10468) was installed in the sample beam path to control the wavefront of the sample wave. This wave was then reduced in size by a factor of 444 through a condenser lens and delivered to a sample located at the focal plane of the condenser lens. The pixel size of the SLM was 20 × 20 μm^2^, and typically 100 × 100 pixels were used for the illumination, which corresponds to 5 × 5 μm^2^ at the sample with each pixel measuring 45 × 45 nm^2^. For a sample, a 100 nm-thick Au film was coated on standard slide glass, and nanostructures were fabricated using a focused ion beam. The sample was placed in such a way that the coated layer faced the condenser lens, with no immersion medium inserted between the sample and the lens. The SPPs were generated at the metal/air interface, traveled through the metal layer to the slide glass, and were captured by an objective lens (Nikon Achr-Apl, NA = 1.4) via immersion oil. This was possible because the magnitude of the wavevector of the SPPs generated at the metal/air interface, k_SPP_ = n_SPP_ × k_0_ with k_0_ = 2π/λ (the wavenumber in free space), is smaller than the maximum wavevector n_g_ × k_0_ that the glass and immersion oil can support. The effective refractive index of SPPs at the metal/air interface is n_SPP_ = 1.057, and the refractive index of both the slide glass and the immersion oil is n_g_ = 1.515. Because the wavevectors of the SPPs remain on the lateral plane (i.e. the x-y plane in Fig. 1), the propagation angle θ_SPP_ of the SPPs with respect to the optical axis in the glass layer is given by k_SPP_ = n_g_k_0_sinθ_SPP_. This means that the SPPs appeared as a circular ring on the back focal plane of the objective lens. In addition to the SPPs, far-field waves were scattered by the nanostructures and subsequently captured by the slide glass. These far-field waves can cover the entire circular area on the back focal plane of the objective lens. However, the high spatial frequencies of these scattered far-field waves tend to be weaker than their low frequency components. For this reason, a clear ring pattern associated with the SPPs was clearly visible in the background of the far-field waves. We placed a circular plate (BB) at plane conjugate to the back aperture of the objective lens to block far-field waves whose lateral wavevector is smaller than k_SPP_. A tube lens (TL) was positioned downstream to form an image of the transmitted SPPs in the camera. In this way, the light wave measured at the camera is made proportional to the SPPs generated at the air/metal interface. A reference wave was introduced to the camera via a beam splitter (BS) to form an interference image, from which we obtained the amplitude and phase maps of the transmitted SPPs.

### Analytical approach to simple double-slit structures

**Figure 2:**
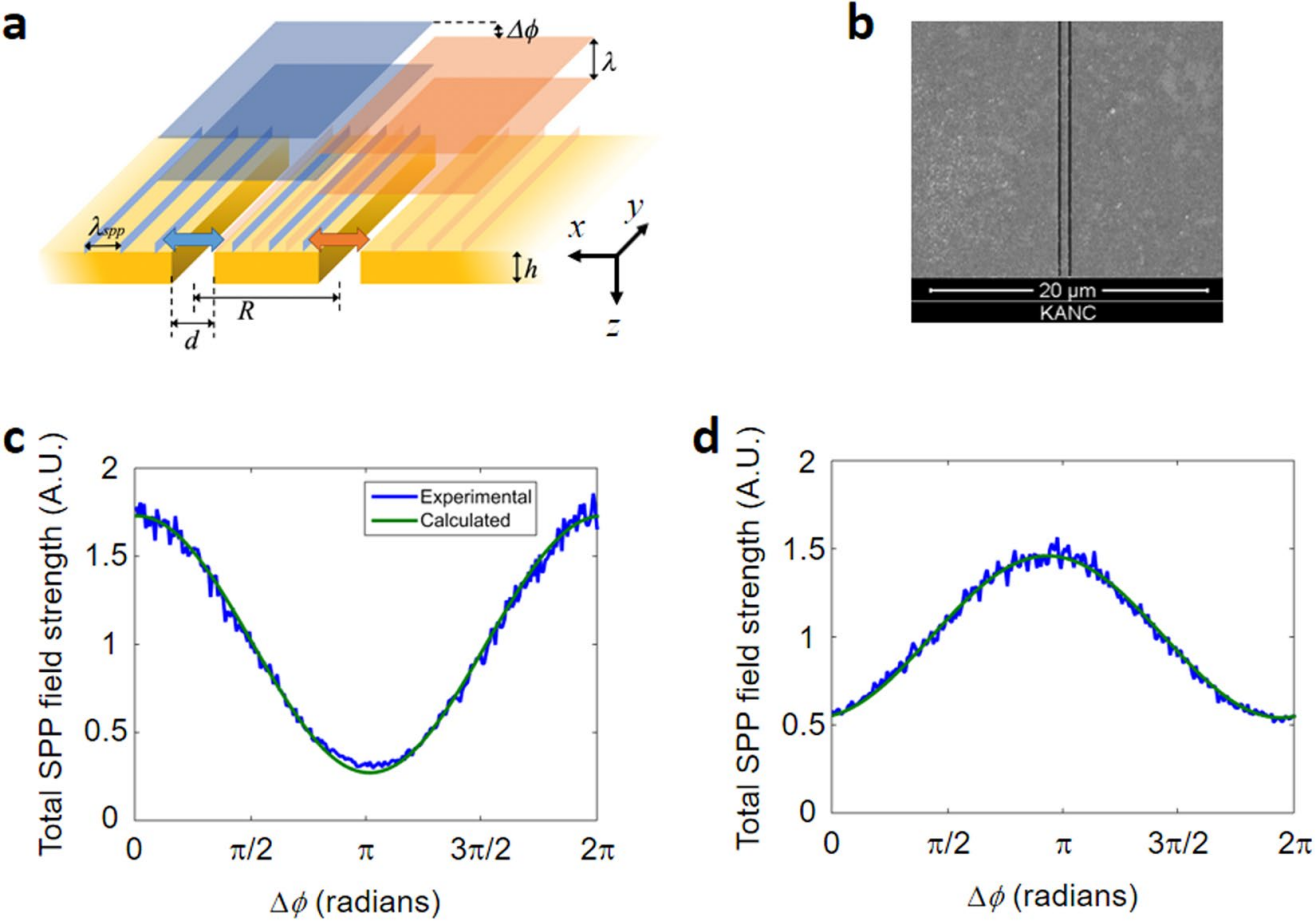
The relationship between total SPP field strength and the phase difference of the incident wave at the two slits. (**a**) Schematic diagram for the generation of SPPs. *R* is the distance between the centers of the two slits, *h* is the thickness of the Au film, and *d* is the width of the individual slits. Δ*φ* is the phase difference between two plane waves incident to the left- and right-hand slits, indicated by the planar squares in blue and orange, respectively. *λ* is the wavelength of the incident light, and *λ*_spp_ is the wavelength of the SPPs at the metal/air interface. (**b**) Sample image taken via focused ion beam scanning. (**c** and **d**) Total SPP field strength vs. Δ*φ* for *R*_1_ = 550 nm and *R*_2_ = 850 nm, respectively. Experimental results are shown in blue and the fitted curves based on theoretical predictions (Eq. 6) in green.

To validate the proposed eigenchannel coupling approach to maximize the generation of SPPs, we first considered simple double-slit structures (Fig. 2a) that the extended CMM could handle. For these simple structures, the incidence wave that either maximizes or minimizes the total field strength of SPPs defined by the absolute square of the electric field of SPPs can be calculated. Let us assume that the two slits are separated by distance *R* and the width of an individual slit is *d*. The generation of SPPs for this double-slit structure can be described using analytic theory based on the CMM. In an ordinary CMM, the incident wave is assumed to be Gaussian with a flat wavefront. We extended this original theory to add spatial wavefront control of the incident wave. The interaction between the two modes from two respective slits can be described by the following set of equations (see the Supplementary Information for the detailed derivation of these equations):

1 $$({G}_{S}-{\rm{\Sigma }}){E}_{l}+{G}_{D}(R){E}_{r}-{G}_{\nu }{E}_{l}^{^{\prime} }={E}_{l}^{in}$$

2 $$({G}_{S}-{\rm{\Sigma }}){E}_{l}^{^{\prime} }+{G}_{D}(R){E}_{r}^{^{\prime} }-{G}_{\nu }{E}_{l}=0$$

3 $$({G}_{S}-{\rm{\Sigma }}){E}_{r}+{G}_{D}(R){E}_{l}-{G}_{\nu }{E}_{r}^{^{\prime} }={E}_{r}^{in}$$

4 $$({G}_{S}-{\rm{\Sigma }}){E}_{r}^{^{\prime} }+{G}_{D}(R){E}_{l}^{^{\prime} }-{G}_{\nu }{E}_{r}=0$$

Eqs (1) and (2) are derived from the boundary conditions at the entrance and exit planes of the metal layer, respectively, for the left-hand slit. Likewise, Eqs. (3) and (4) are derived for the right-hand slit. ${E}_{l}^{in}$ and ${E}_{r}^{in}$ refer to the electric fields of the incident waves at the left- and right-hand slits, respectively. *E*_*l*_ and *E*_*r*_ are the electric fields at the left- and right-hand slits, respectively, for the entrance plane, and ${E}_{l}^{\text{'}}$ and ${E}_{r}^{\text{'}}$ are for the exit plane. *G*_*D*_ is the mode-mode propagator, which represents the coupling between the fundamental modes of the two slits. Therefore, it depends on the separation *R*. *G*_*S*_, *G*_*ν*_, and Σ are determined by the waveguide structure of the fundamental mode. *G*_*S*_ represents the self-coupling of each slit, *G*_*ν*_ describes the coupling of the electromagnetic field from the opposite side of the slit, and Σ relates to the waves bouncing back and forth inside the slit.

This set of equations can be rewritten in the form of the following matrix equation:

5 $$(\begin{array}{c}{E}_{l}\\ {E}_{r}\end{array})=(\begin{array}{cc}{t}_{11} & {t}_{12}\\ {t}_{21} & {t}_{22}\end{array})(\begin{array}{c}{E}_{l}^{in}\\ {E}_{r}^{in}\end{array}).$$

In this equation, *t*_*ij*_ is the element of the transmission matrix connecting the far-field input to the SPPs generated at the entrance plane. Each matrix element can be written using the coefficients in Eqs. (1)–(4). To account for the wavefront shaping of the incident wave, we set the electric field of the incident wave as ${E}_{r}^{in}={E}_{l}^{in}{e}^{i{\rm{\Delta }}\varphi }$, where Δ*φ* is the phase difference between the left- and right-hand slits. Our interest lies in finding the conditions under which the generation of the SPPs at the entrance plane of the two slits is maximized. From the transmission matrix, we can obtain the total transmitted field strength of the SPPs as a function of Δ*φ*:

6 $${|{E}_{l}|}^{2}+{|{E}_{r}|}^{2}\propto 1+a{e}^{-\xi R}\,\cos ({k}_{spp}R)\cos \,{\rm{\Delta }}\varphi .$$

Here, *a* and *ξ* are the coefficients derived from Eq. (5). (See Supplementary Information for a detailed derivation.) These are related to the coupling strength between the two slits and thus the separation *R*. The total field strength oscillates with Δ*φ* and the contrast of the oscillation is given by $a{e}^{-\xi R}\,\cos ({k}_{spp}R)$. Therefore, the Δ*φ* that maximizes the total field strength of SPPs is either 0 or π depending on the sign of $\cos ({k}_{spp}R)$. In other words, the separation *R* determines whether the symmetric or anti-symmetric coupling of the incident wave is optimal.

We verified this theoretical prediction by experimentally varying Δ*φ* and measuring the total field strength of the generated SPPs. In this experimental demonstration, a double-slit structure was fabricated on a 100 nm-thick Au film (Fig. 2b). Two different slit separations were prepared, *R*_1_ = 550 nm and *R*_2_ = 850 nm. The width of each slit was *d* = 250 nm, which is narrow enough for only a single mode to exist for each slit. We loaded these two samples into the experimental setup shown in Fig. 1. The SLM was divided into two segments, with one of the segments illuminating the left-hand slit and the other the right-hand slit. A phase retardation Δ*φ* was added to the segment targeting the right-hand slit. While scanning Δ*φ*, we measured the total field strength of the SPPs imaged at the camera. The results are shown as blue curves in Fig. 2(c) and (d). We observed that maximum SPP field strength is observed at Δ*φ* = 0 for *R*_1_ and Δ*φ* = *π* for *R*_2_. Because $\cos ({k}_{spp}{R}_{1})$ > 0 and $\cos ({k}_{spp}{R}_{2})$ < 0, these results are in good agreement with Eq. (6). We can obtain the coefficients *a* and *ξ*by fitting the two curves to Eq. (6) for *R*_1_ = 550 nm and *R*_2_ = 850 nm. The green curves in Fig. 2c and d represent the results of this curve fitting. Overall, it is clear that the extended CMM can accurately predict the incident wave that maximizes the generation of SPPs. However, this analytical approach is limited to well-defined structures, and its extension to more complex structures tends to be extremely difficult because the analytic expressions become too complicated.

### Eigenchannel coupling approach for a double-slit structure

**Figure 3:**
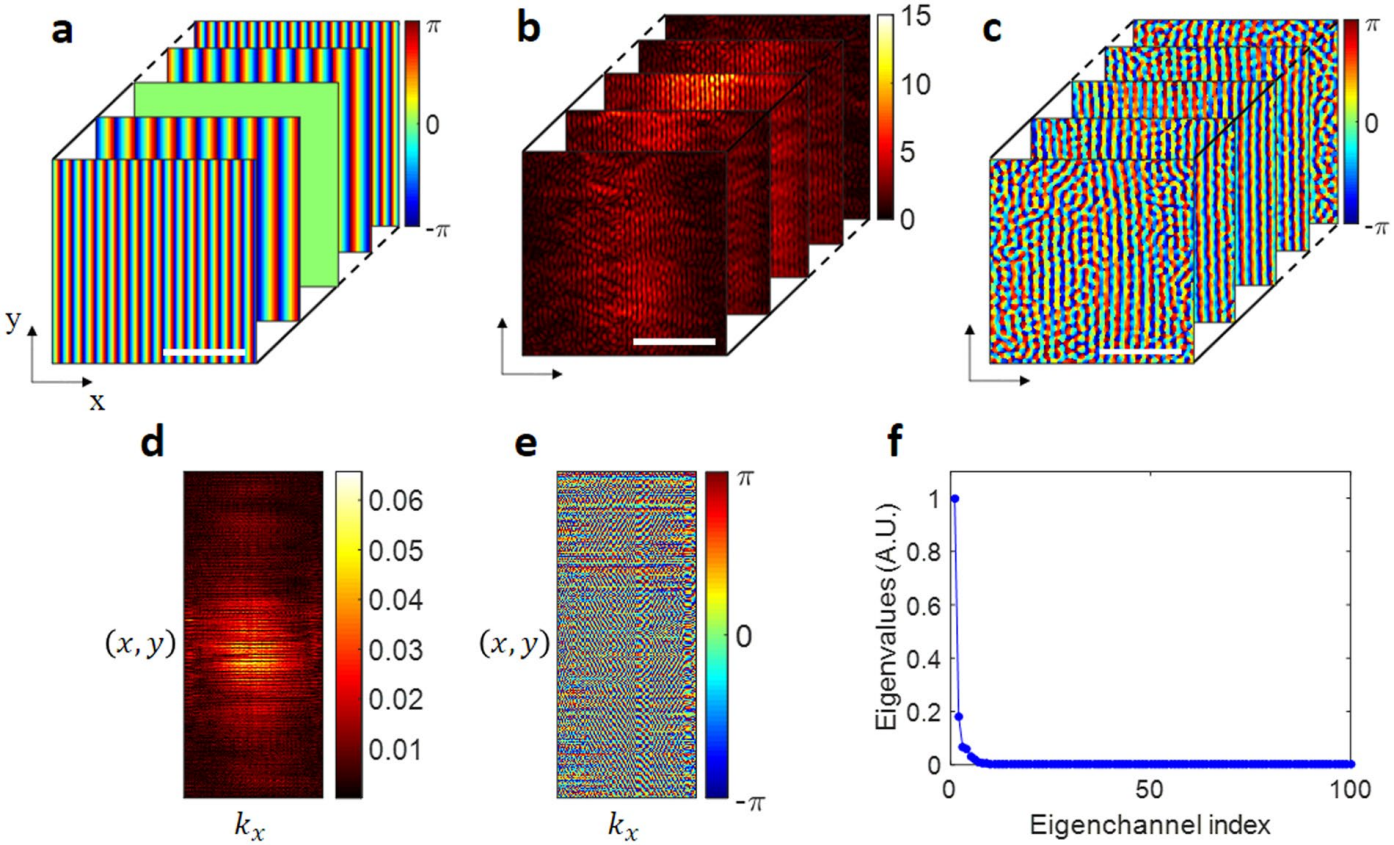
Construction of a transmission matrix and identification of its singular values. (**a**) Phase maps of the incident waves. (**b** and **c**) Amplitude and phase maps, respectively, of the SPPs generated by the incident waves. Scale bars: 5 μm. (**d** and **e**) Amplitude and phase components of the transmission matrix constructed from (**b** and **c**). Each complex field map formed by (**b** and **c**) was converted to a column in (**d**). (**f**) Singular values from the transmission matrix. The colored bars in (**a,c**, and **e**) indicate the phase in radians, and that in (**b** and **d**) the amplitude in arbitrary units.

The mathematical process for finding the Δ*φ* that maximizes the total field strength of SPPs in Eq. (6) is equivalent to finding the eigenchannel in a transmission matrix that has the highest singular value. In this section, we confirmed the validity of our experimental approach by comparing it with the extended CMM discussed in Section III. We first constructed a transmission matrix using the experimental setup shown in Fig. 1. Because the double slits varied in structure only along the *x*-direction, the input basis of the transmission matrix was covered along the same direction. In other words, we scanned the angle of the incident wave only along the *x*-direction (Fig. 3a). The number of incidence channels was 100, a number which was selected based on the width of the illumination in *x* and the numerical aperture of the objective lens. Representative amplitude and phase maps for the SPPs recorded for various incident waves are shown in Fig. 3b and c. A transmission matrix *t*(*x*,*y*;*k*_*x*_) with *k*_*x*_ = *k*_0_*sinθ*_*x*_ (*θ*_*x*_ is the angle of illumination) was constructed from these measurements (Fig. 3d and e). We then performed a singular value decomposition, *t* = *UτV* ^+^, where *U* and *V* are unitary matrices containing the eigenvectors for the SPPs and the far-field input, respectively, *τ* is a non-negative diagonal matrix, and + is the conjugate transpose of the matrix. The diagonal element *λ*_*i*_ in *τ* is a singular value corresponding to the conversion efficiency from far-field waves to SPPs, where *i* is the eigenchannel index, and these elements are arranged in descending order so that *λ*_*i*_ > *λ*_*j*_ if *i* < *j*. Column ***v***_*i*_ of *V* corresponds to the eigenchannels for the incident wave associated with *λ*_*i*_. Because the two small slits each had only a single fundamental mode, there were only two meaningful singular values (Fig. 3f). Other singular values were too small to be meaningful because they were at the noise level set in the absence of the double-slit structure.

**Figure 4:**
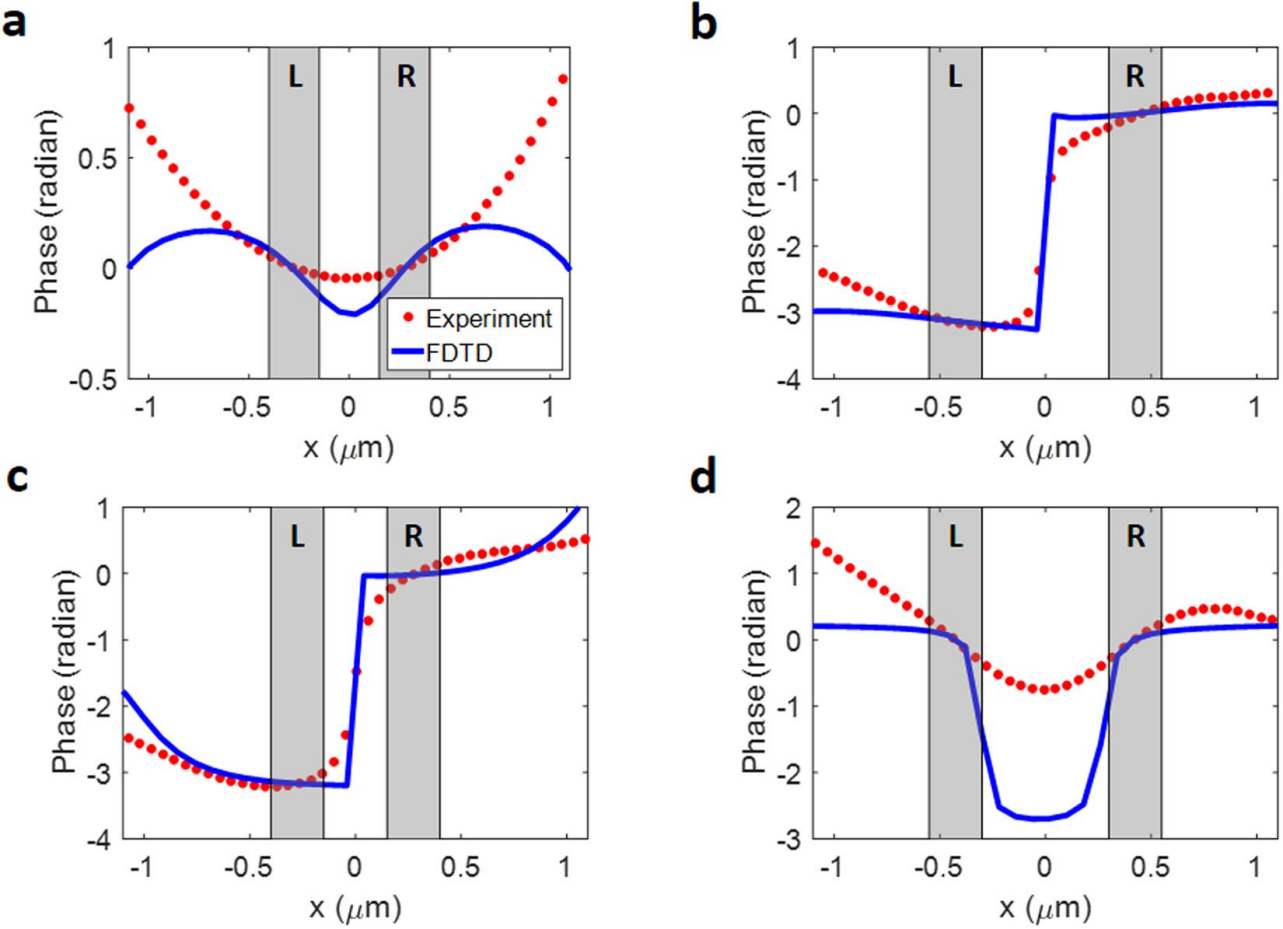
Phase profiles of the input eigenchannels based on slit separation. (**a**) and (**b**) the first eigenchannels for *R*_1_ = 550 nm and *R*_2_ = 850 nm, respectively. (**c** and **d**) the same as (**a**) and (**b**), respectively, but for the second eigenchannels. The red dots show the phase of the input eigenchannels obtained from the experiment and the blue curves show the phase of the input eigenchannels calculated using the FDTD method. The positions of the slits are indicated as gray columns. L and R stand for the left- and right-hand slits, respectively.

The phase profiles of input eigenchannels reconstructed from the column vectors of *V* are plotted in Fig. 4. Figure 4a and b present the phase profile of the eigenchannel with the highest singular value along the *x*-direction for *R*_1_ = 550 nm and *R*_2_ = 850 nm, respectively. For *R*_1_, the phase values at the left- and right-hand slits were almost the same, i.e. Δ*φ* ≈ 0. On the other hand, the phase values at the slits differed by almost π for *R*_2_. These results agree with the predictions made by both the extended CMM and the direct phase modulation experiment presented in section III. In addition, we performed numerical simulations using the FDTD method^23,29^ for the same sample geometry (the green curves in Fig. 4a and b) and confirmed the experimental observations. We also plotted the eigenchannels with second largest singular values (Fig. 4c and d) in which the total field strength of SPP is minimized. In these cases, Δ*φ* ≈ *π* for *R*_1_ and 0 for *R*_2_, which also agree with the theoretical prediction. These results confirm that the identification of eigenchannels is a systematic approach to determining the optimal coupling conditions for the generation of SPPs.

### Transmission matrix approach for arbitrary structures

For simple structures like double slits, the extended CMM and the eigenchannel-coupling approach work equally well. As target structures become complex, however, it is more difficult to analytically calculate the appropriate incident wave. In addition, the designed structures may have defects that the theoretical modeling cannot account for. Despite this, it is possible in an experiment to construct the matrix regardless of the shape of the structures. Moreover, the computation of this matrix and its coupling to eigenchannels are experimentally straightforward. Therefore, the eigenchannel approach can be extended to arbitrarily complex structures.

**Figure 5:**
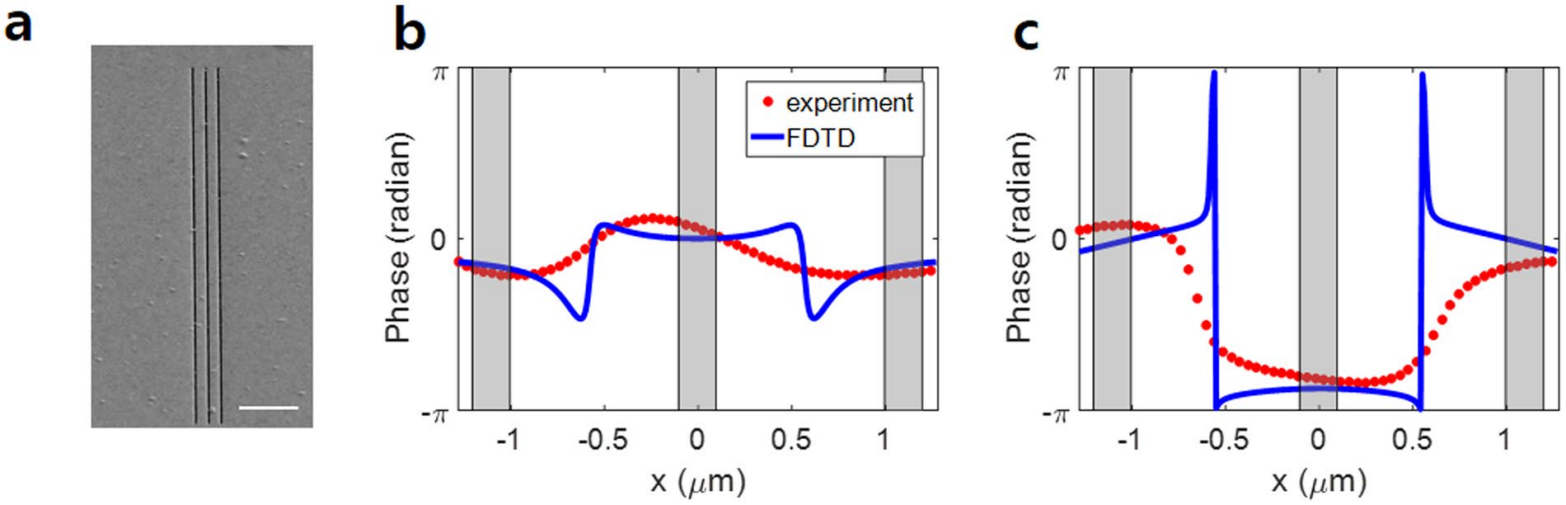
Eigenchannels of triple slits fabricated on a thin Au film. (**a**) Sample image measured from focused ion beam scanning. Scale bar: 5 μm. (**b)** Phase profiles of the first input eigenchannel measured by the experiment (red circular dots) and calculated using the FDTD method (solid blue curve). (**c)** The same as (**b)** but for the third eigenchannel. The shaded areas in gray indicate the location of the slits.

As a test sample, we fabricated triple slits on a 100 nm-thick Au film (Fig. 5a). The distance between neighboring slits was R = 1.0 μm, and the width of the individual slits was 200 nm. Although it is possible to analytically calculate the incident wave that maximizes the generation of SPPs for this triple-slit structure, this requires lengthy calculations. Instead, we used the FDTD method to calculate the transmission matrix. Because the three slits only had a single fundamental mode, there were three singular values, *λ*_1_ > *λ*_2_ > *λ*_3_ and three associated input eigenchannels, ***v***_1_,***v***_2_, and ***v***_3_. The phase profiles of ***v***_1_ and ***v***_3_ are displayed as the blue curves in Fig. 5b and c, respectively. Because the distance between the neighboring slits was close to 2 *λ*_SPP_, i.e. the integer multiple of the wavelength, we can expect symmetric coupling to maximize the generation of SPPs. The phase profile of ***v***_1_ is in agreement with this prediction because the phase values of the first input eigenchannel at the three slits were almost the same (Fig. 5b). In contrast, anti-symmetric coupling is expected to minimize the generation of SPPs, and indeed the phase difference between the neighboring slits was close to *π* for ***v***_3_ (Fig. 5c).

We made experimental measurements of the transmission matrix for the triple-slit structure and obtained its singular values and input eigenchannels. The red circular dots in Fig. 5b and c show the phase profiles of the experimentally acquired ***v***_1_ and ***v***_3_, respectively. We observed that the phase differences among the different slits were in excellent agreement with the prediction made from FDTD simulations. For this three-slit sample, we analyzed in the FDTD simulations the absolute coupling efficiency in terms of the field strength of the light waves. For a normally incident plane wave of unity amplitude, the average field strength of SPPs was calculated to be about 0.063. On the other hand, the field strength of the SPPs generated by the eigenchannel coupling was about 0.25, i.e. four times larger than that of the normally incident plane wave.

**Figure 6:**
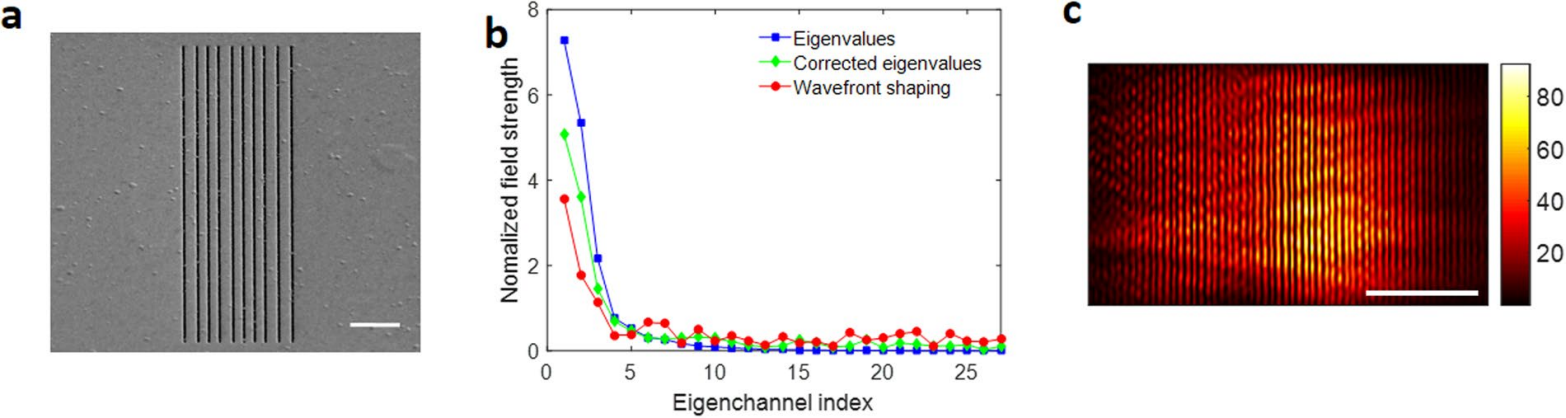
Maximal coupling to SPPs for a disordered array of slits. (**a**) Sample image derived from focused ion beam scanning. Scale bar: 5 μm. (**b**) Total field strength of the SPPs generated by the coupling of the incident waves to the eigenchannels (red circular dots). The SPP field strength was normalized by that of the normally incident plane wave. Eigenvalue distribution of the measured transmission matrix (blue square dots) and the corrected eigenvalues accounting for the phase-only wavefront control (green diamond dots) are plotted for comparison. Eigenvalues were normalized by the field strength of the SPPs for the normally incident plane wave. (**c**) SPP field strength map when the incident wave was experimentally coupled to the first eigenchannel. Scale bar: 5 μm.

Finally, we tested a sample composed of multiple slits with random separations (Fig. 6a). For this type of sample, it is almost impossible to predict the incident wave that can maximize the generation of SPPs. The FDTD calculation may not be applicable either because the fabricated structure has defects that cannot be quantified for the input to the FDTD simulation. As with the double- and triple-slit structures, we experimentally measured the transmission matrix and identified the eigenchannels. After shaping the incident wavefront to that of the eigenchannels, we measured the total field strength of the SPPs generated on the surface of the metal (circular dots in Fig. 6b). In comparison with the normally incident plane waves, we observed that the SPPs were close to four times stronger. As a point of reference, we plotted the eigenvalue distribution calculated from the measured transmission matrix and the corrected eigenvalues after accounting for the phase-only control of SLM. Although the coupling efficiency obtained by the wavefront shaping was observed to be lower, the overall trend was similar to the expectations based on the transmission matrix. These observations confirmed that our experimental approach is appropriate for the maximal generation of SPPs using an arbitrary structure.

## Conclusion

For arbitrarily complex nanostructures, we have demonstrated that the coupling efficiency of far-field waves to SPPs can be close to 4 times higher when shaping the wavefront of the incident wave to eigenchannels identified from the experimentally measured transmission matrix. The proposed method was validated for a simple double-slit structure by comparing it with the theoretical calculation based on coupled mode method. As the complexity of plasmonic devices are expected to increase to allow the integration of various functionalities, the fall in conversion efficiency of far field waves to SPPs will be a potential problem. Our method of efficiently coupling far-field waves to SPPs for any arbitrarily complex nanostructure may relive the constraints in design freedom and expedite the development of multi-functional plasmonic devices. For example, the eigenchannels of a given complex device will depend on the wavelength. Wavefront shaping to the eigenchannel for one wavelength will be just random inputs for the other wavelengths. This opens the possibility of implementing highly scalable wavelength-dependent switching devices. Another example is to exploit mode-dependent eigenchannels. Among the all the plasmonic modes, we can choose a subset of the modes and maximize their field strength. In this way, we address many subsets of plasmonic modes to which multiple plasmonic devices are connected.

## Electronic supplementary material

Supplementary Information
