# Author Correction: Maximizing energy coupling to complex plasmonic devices by injecting light into eigenchannels

**DOI:** 10.1038/s41598-018-23477-1

**Published:** 2018-04-05

**Authors:** Yonghyeon Jo, Wonjun Choi, Eunsung Seo, Junmo Ahn, Q-Han Park, Young Min Jhon, Wonshik Choi

**Affiliations:** 1 Center for Molecular Spectroscopy and Dynamics, Institute for Basic Science, Seoul, 02841 Korea; 2 Department of Physics, Korea University, Seoul, 02841 Korea; 3 Sensor System Research Center, Korea Institute of Science and Technology, Seoul, 02792 Korea

Correction to: *Scientific Reports* 10.1038/s41598-017-10148-w, published online 29 August 2017

The Acknowledgements section in this Article was omitted. The Acknowledgements section should read:

“This research was supported by IBS-R023-D1 and the Global Frontier Project (2014M3A6B3063710) through the National Research Foundation of Korea (NRF) funded by the Ministry of Science, ICT and Future Planning. It was also supported by the Korea Health Technology R&D Project (HI14C0748) funded by the Ministry of Health and Welfare, Republic of Korea.”

