# Supplementary material for: Maximizing energy coupling to complex plasmonic devices by injecting light into eigenchannels
Source: Sci Rep. 2017 Aug 29;7:9779. doi: 10.1038/s41598-017-10148-w (PMC5574893; doi:10.1038/s41598-017-10148-w)
Supplement: Supplementary file 1 — Supplementary Information [file 41598_2017_10148_MOESM1_ESM.pdf]

Maximizing energy coupling to complex plasmonic devices by injecting light into eigenchannels

Yonghyeon Jo[1,2,*], Wonjun Choi[1,2,*], Eunsung Seo[1,2], Junmo Ahn[1,2,3], Q-Han Park[2], Young Min Jhon[3], and Wonshik Choi[1,2,+]

[1] Center for Molecular Spectroscopy and Dynamics, Institute for Basic Science, Seoul 02841, Korea.
[2] Department of Physics, Korea University, Seoul 02841, Korea.
[3] Sensor System Research Center, Korea Institute of Science and Technology, Seoul 02792, Korea.
[*] These authors contributed equally to this work.
[+] To Whom correspondence and requests for materials should be addressed (email: wonshik@korea.ac.kr).

I. Extended coupled-mode method

In this section, we are going to describe the extend coupled-mode method (ECMM) that is derived from the coupled-mode method (CMM). The CMM is a general framework to analyze the interaction among modes in the nanostructures and it is well described in the previous studies[1]. Therefore, we will briefly introduce the CMM and mainly describe its detailed extension to incorporation the wavefront shaping of the illumination.

Figure S1 is the schematic figure to explain the CMM. Incident light coming from the region I enters region II and exits to region III. The region I and III are filled with the dielectric materials and semi-infinite space. The metal film (region II) has plural holes (or slit) having a waveguide mode. The thickness of the metal film is set *h*.

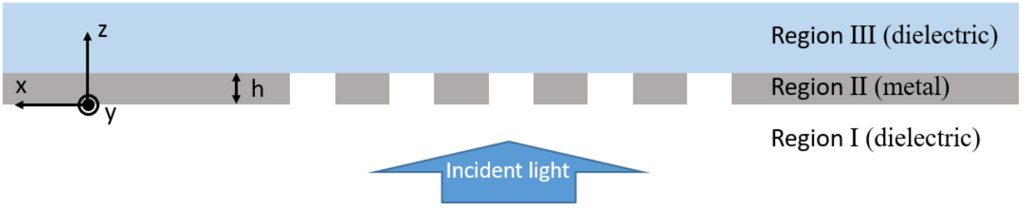

Figure S1. The schematic diagram of the sample.

The below are the equations resulting from the CMM, and they can be a good starting point to explain the ECMM.

$$(G_{\alpha\alpha} - \mathbf{\Sigma}_\alpha)E_\alpha + \sum_{\beta\neq\alpha} G_{\alpha\beta}E_\beta - G_\alpha^\nu E'_\alpha = I_\alpha \quad \text{(S1a)}$$

$$\left(G'_{\alpha\beta} - \mathbf{\Sigma}_\alpha\right)E'_\alpha + \sum_{\beta\neq\alpha} G'_{\alpha\beta}E'_\beta - G_\alpha^\nu E_\alpha = 0 \quad \text{(S1b)}$$

The equation S1a and S1b are for the boundary matching at $z = 0$ and $z = h$, respectively. Here the terms in equations are defined as follows.

$$I_\alpha = 2\frac{Y_{\mathbf{k}_\parallel^0\sigma^0}}{f^+_{\mathbf{k}_\parallel^0\sigma^0}}\left\langle\alpha\middle|\mathbf{k}_\parallel^0\sigma^0\right\rangle,$$

$$G_{\alpha\beta} = \sum_{\mathbf{k}_\parallel\sigma} Y_{\mathbf{k}_\parallel\sigma}\frac{1}{f^+_{\mathbf{k}_\parallel\sigma}}\left\langle\alpha\middle|\mathbf{k}_\parallel\sigma\right\rangle\left\langle\mathbf{k}_\parallel\sigma\middle|\beta\right\rangle,$$

$$G'_{\alpha\beta} = \sum_{\mathbf{k}_\parallel\sigma} Y'_{\mathbf{k}_\parallel\sigma}\frac{1}{f'^+_{\mathbf{k}_\parallel\sigma}}\left\langle\alpha\middle|\mathbf{k}_\parallel\sigma\right\rangle\left\langle\mathbf{k}_\parallel\sigma\middle|\beta\right\rangle, \quad \text{(S2)}$$

$$\mathbf{\Sigma}_\alpha = Y_\alpha\frac{f_\alpha^- e^{-ik_z^\alpha h} + f_\alpha^+ e^{ik_z^\alpha h}}{f_\alpha^+ f_\alpha^+ e^{ik_z^\alpha h} - f_\alpha^- f_\alpha^- e^{-ik_z^\alpha h}}, \quad \text{and} \quad G_\alpha^\nu = \frac{2Y_\alpha}{f_\alpha^+ f_\alpha^+ e^{ik_z^\alpha h} - f_\alpha^- f_\alpha^- e^{-ik_z^\alpha h}} .$$

The I_α represents the coupling between the incident field and the waveguide mode α. The E_α and E'_α are the electric field at the input and output interfaces of the holes, respectively. $G_{\alpha\beta}$ and $G'_{\alpha\beta}$ represent the coupling between the waveguide modes α and β of two different holes at the $z = 0$ and $z = h$, respectively. Therefore, the $G_{\alpha\alpha}$ and $G'_{\alpha\alpha}$ can be considered the self-interaction coupling of the waveguide mode α at the $z = 0$ and $z = h$, respectively. The $\mathbf{\Sigma}_\alpha$ is the net of the waveguide mode α between up (positive z) and down (negative z) directions, and the G_α^ν the effect of the field on the one side to the field on the other side in waveguide with mode α.

In general, two equations are needed to obtain the E_α and E'_α for one waveguide mode α. Therefore, we need the $2N$ equations for N holes. In our study, the metal film (region II) has only two slits with same width, and the width of slits are very narrow such that it is safe to assume that there is a single mode per each slit. Furthermore, if the incident field is normal to the metal film, only two equations are enough because of the symmetry of the given system. However, we inject different fields into two slits in our study, and the following four equations are required as a consequence.

$(G_S - \Sigma)E_l + G_D(R)E_r - G_v E_l' = E_l^{in}$ (S3)

$(G_S' - \Sigma)E_l' + G_D'(R)E_r' - G_v E_l = 0$

$(G_S - \Sigma)E_r + G_D(R)E_l - G_v E_r' = E_r^{in}$

$(G_S' - \Sigma)E_r' + G_D'(R)E_l' - G_v E_r = 0$

Here, the subscript l and r indicate the left and right slits, respectively. In the main text, we assumed $G_S' = G_S$ and $G_D' = G_D$ as the refractive indices of the materials in the region I and II are the same.

II. Derivation of a transmission matrix

We can rewrite Eq. S3 as the following matrix equation.

$$\begin{pmatrix} a & G_D(R) & b & 0 \\ G_D(R) & a & 0 & b \\ b & 0 & a & G_D(R) \\ 0 & b & G_D(R) & a \end{pmatrix} \begin{pmatrix} E_l \\ E_r \\ E_l' \\ E_r' \end{pmatrix} = \begin{pmatrix} E_l^{in} \\ E_r^{in} \\ 0 \\ 0 \end{pmatrix} \qquad \text{(S4).}$$

Here $a = (G_S - \Sigma)$ and $b = -G_v$, and $G_D(R) = L(R)e^{ik_{spp}R}$, where $L(R)$ means the metallic loss decaying exponentially. The higher order terms of $G_D(R)$ is ignored in this calculation as they are extremely smaller than the linear term. From this equation, we obtain E_l and E_r, which are SPP field resided between the region I and II (S5).

$$E_l \cong \frac{a^3E_l^{in} - ab^2E_l^{in} - a^2G_D(R)E_r^{in} - G_D(R)b^2E_r^{in}}{a^4 + b^4 - 2a^2b^2}$$

$$E_r \cong \frac{-a^2G_D(R)E_l^{in} - G_D(R)b^2E_l^{in} + a^3E_r^{in} - ab^2E_r^{in}}{a^4 + b^4 - 2a^2b^2}. \qquad \text{(S5)}$$

For the sake of simplicity, we replace the common denominator of E_l and E_r, $(a^2 - b^2)^2$, with $1/A$ in Eq. (S6).

$$E_l \cong A(a^3 - ab^2)E_l^{in} - AG_D(R)(a^2 + b^2)E_r^{in}$$

$$E_r \cong -AG_D(R)(a^2 + b^2)E_l^{in} + A(a^3 - ab^2)E_r^{in}. \qquad \text{(S6)}$$

Finally, we construct the transmission matrix introduced in our main manuscript.

$$\begin{pmatrix} E_l \\ E_r \end{pmatrix} = \begin{pmatrix} t_{11} & t_{12} \\ t_{21} & t_{22} \end{pmatrix} \begin{pmatrix} E_l^{in} \\ E_r^{in} \end{pmatrix} \quad \text{(S7)}$$

Here, the elements of the transmission matrix are as below,

$t_{11} = t_{22} = A(a^3 - ab^2)$ and $t_{12} = t_{21} = -AG_D(R)(a^2 + b^2)$.

III. Finding an incident wave maximizing SPPs

In this section, we derive the equation (6) in main manuscript. To this end, we replace E_r^{in} and $G_D(R)$ in the equation (S6) with $E_l^{in} e^{i\Delta\phi}$ and $L(R)e^{ik_{\mathrm{spp}}R}$, respectively, which was mentioned in the main manuscript and supplementary section II. Therefore, the rewritten equations are as below,

$$E_l \cong A\left(a(a^2 - b^2) - L(R)(a^2 + b^2)e^{i(k_{spp}R + \Delta\phi)}\right) E_l^{in}$$

$$E_r \cong -A\big(L(R)e^{ik_{\mathrm{spp}}R}(a^2 + b^2) - a(a^2 - b^2)e^{i\Delta\phi}\big)E_l^{in}. \quad \text{(S8)}$$

To obtain the total intensity of SPPs we take the absolute squares of the E_l and E_r and obtained the summation of the two.

$$|E_l|^2 + |E_r|^2 \cong 2\left|AE_l^{in}\right|^2 \left(a^2(a^2 - b^2)^2 - 2aL(R)(a^4 - b^4)\cos\big(k_{\mathrm{spp}}R\big)\cos(\Delta\phi)\right) \quad \text{(S9)}$$

Here, we assumed that $L(R) \propto e^{-\xi R}$ and ignored $L^2(R)$ as was done in Section II. Finally, the total intensity is simplified to the Eq. (6) in the main manuscript.

Reference

1. de Leon-Perez, F.; Brucoli, G.; Garcia-Vidal, F. J.; Martin-Moreno, L. *New J Phys* **2008,** 10.
